# The Impact of the Current SARS-CoV-2 Pandemic on Neonatal Care

**DOI:** 10.3389/fped.2020.00247

**Published:** 2020-04-30

**Authors:** Juan Arnaez, Maria Teresa Montes, Nuria Herranz-Rubia, Alfredo Garcia-Alix

**Affiliations:** ^1^Department of Neonatology, Burgos University Hospital, Burgos, Spain; ^2^NeNe Foundation, Madrid, Spain; ^3^Department of Neonatology, Hospital Universitario La Paz, Madrid, Spain; ^4^Department of Neonatology, Hospital Sant Joan de Deu, Barcelona, Spain; ^5^Department de Cirugia i Especialitats Medicoquirúrgiques, Universitat de Barcelona, Barcelona, Spain; ^6^Instituto de Recerca Sant Joan de Deu, Hospital Sant Joan de Deu, Barcelona, Spain

**Keywords:** SARS-CoV-2, neonate, perinatal care, family-centered care, pandemic, moral distress, covid-19

The current 2019 coronavirus disease (SARS-CoV-2) pandemic has turned out to be the largest and most pervasive health emergency worldwide. Although novel coronavirus disease (Covid-19) is insistently attacking the adult population, contingency plans are impacting all areas of medicine worldwide. In particular, puerperants, parents of newborns, and infants are becoming infected with severe consequences on neonatal assistance.

At present, there is no definitive evidence that SARS-CoV-2 can be transmitted transplacentally (1–5), and there are no virus detection reports of SARS-CoV-2 in amniotic fluid or placenta in infected pregnant women (6). However, data are scarce on whether early-stage fetal infection can lead to teratogenic effects.

An encouraging fact in neonatal medicine is that the horizontally infected neonates reported to date have shown a mild clinical profile and good outcome (7, 8). Nevertheless, the current SARS-CoV-2 outbreak is bringing about considerable changes in the care policy of neonatology units that affect not only infants with SARS-Cov-2 infection and infants of infected parents, but also the care offered to other admitted patients (9, 10). These changes mainly impact several key points: (1) the organization and workflow of the neonatal unit, (2) parent-infant bonding and family-centered care, and (3) stress-related consequences in health professionals (Figure 1).

**Figure 1:**
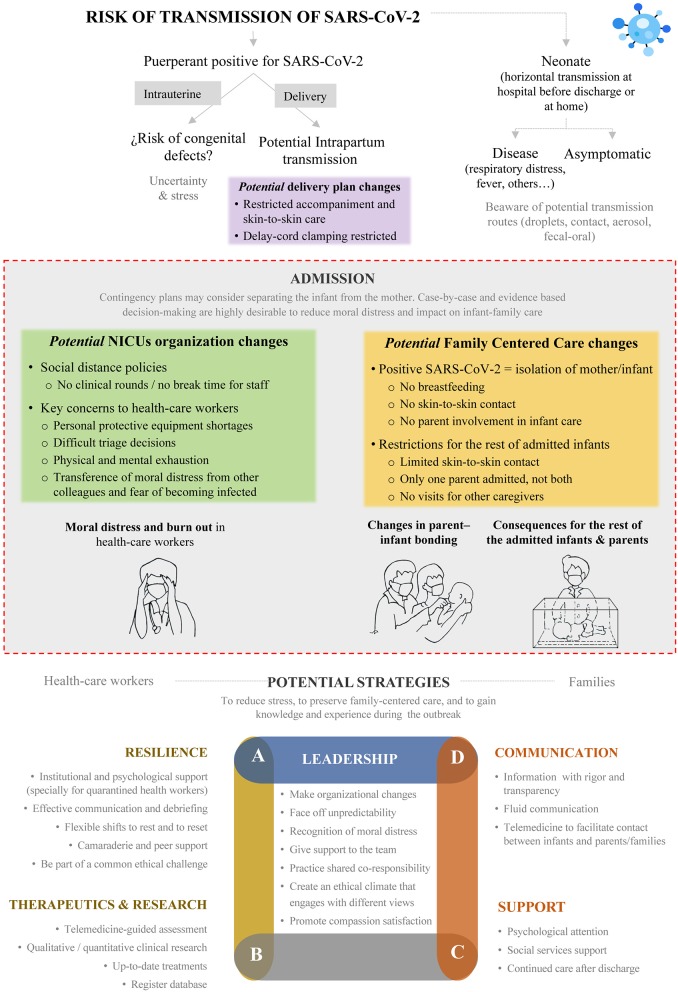
Potential consequences of SARS-CoV-2 pandemic on neonatal care.

During this crisis, neonatal units, as most medical hospital divisions, have needed to implement major changes in their daily workflow. This pandemic has brought about health-worker shortages as staff become infected or replaced in other positions, and thus organizing shifts to ensure quality assistance has become difficult and unpredictable.

Further, pandemic outbreaks bring stress upon health-care workers due to the shortage of medical resources, overwork with long shifts and restrictions on socialization, and the pain of losing colleagues or becoming infected and possibly infecting families. In addition, we should note the moral distress and its effects experienced by health professionals when they are unable to act according to the evidence and their deeply-held convictions concerning family care because of limitations beyond their control.

Well-designed actions that encourage stress reduction, provide psychological support, and promote resilience can help make the day-to-day in neonatal units less stressful. In this situation, perspectives to recognize and mitigate moral distress are necessary. Strategies such as identification the most vulnerable professionals as well as the senior experts, debriefing together about ethically challenging clinical cases, effective communication within the team, accurate guidelines to be followed, and flexibility to facilitate health workers leadership to develop their work efficiently, should help deal with such difficulties and gain moral comfort (11). In this sense, clear and sensitive leadership, interdisciplinary collaboration and mutual support to achieve common goals are essential. Due to the high reported prevalence of psychological distress in quarantined health workers, institutional support for them is essential to facilitate their return to work and to provide psychological assistance if necessary (12).

The family-centered care model has been incorporated into neonatal units based on the ethics of care and scientific evidence that suggests that in order to promote correct neurodevelopment and achieve the best health outcomes of the family unit it is crucial to establish an environment that promotes healing. This model effectively encourages parent-infant bonding in order to improve the ability to provide health throughout development. The family and particularly parents, play an active role as primary caregivers of their child, and responsibility in making decisions is shared between health professionals and the families of infants. Being able to exercise this role of primary caregivers brings benefits in the emotional health of parents that have a positive impact on babies in the medium and long term (13). In addition, promoting this task to parents will also support professional's well-being to better cope with the current pandemic scenario.

Contingency plans during pandemic outbreaks may directly clash with this model, largely due to isolation recommendations. Initial recommendations supported changes to delivery plans by introducing restrictions on early skin-to-skin contact, the presence of the father at childbirth, and late-cord clamping (14, 15). In addition, infants born to infected mothers as well as newborns with confirmed SARS-CoV-2 infection should be separated and isolated in an individual room with specific air handling and the use of protective equipment.

However, current recommendations are being modified on a case-by case basis accounting for the disease severity, illness symptoms, and results of laboratory testing for the virus. In mothers in good clinical condition the separation of the binomial mother-child pair might be not recommended, as long as precautionary measures can be guaranteed to avoid contagion such as using a facemask, practicing hand, and breast hygiene before each feeding, and maintaining a safe distance of two meters. Likewise, infected neonates undergo recommendations that vary from isolated admission without caregivers to strategies adapted to the clinical situation of the baby, but with the accompaniment of their parents (16, 17).

Apart from physiological benefits for mothers and infants, breastfeeding also helps the mother to better cope with the stress of hospitalization, participate directly in the care of the baby, connect emotionally, and facilitate the construction of the maternal role. Even though to date no viral load has been isolated in breast milk and there are no major reasons to avoid breastfeeding in the infants of infected mothers, recommendations for restriction are shared (10, 14). To date, International guidelines advise that breastfeeding should continue, whether or not the lactating parent has SARS-CoV-2 infection, with appropriate precautions (18, 19). Pasteurized donated milk (milk bank) is a crucial resource for intensive care infants whose mothers are unable to provide their own milk temporarily. Interruption of feeding with donated breast milk, particularly in very premature or very low birth weight infants, increases the risk of necrotizing enterocolitis in these children, hence it is considered a major health intervention in these patients. However, in the current situation, most potential donors have to stay home due to general confinement, and given the shortage of reserves, milk bank may need to be prioritized for preterm infants younger than 30 weeks of gestational age or weighing <1,500 g at birth whose mother cannot provide her own milk (20). Strategies must be developed to maintain donations and overcome the difficulties of confinement over milk banks.

The contingency plans required by the circumstances in the current SARS-CoV-2 outbreak scenario must not let us forget that restrictions on parental contact and interventions in the care of infants may entail costs to the families in addition to the loss of opportunities for the newborn to adapt to the extrauterine environment and advance in neurodevelopment. Restrictions on families are especially relevant in the NICU as they cause emotional disturbance and may profoundly alter the bonding and relationship established with the baby if the resulting emotions continue over time. In this sense, it would be desirable to incorporate parents into contingency plan decisions, such as in the schedule of their presence at the neonatal unit. These compensatory strategies can help to reduce parental discomfort and to encourage parental bonding with the infant that leads to better results and provides some protection against the many challenges that arise during the infant's hospital stay and will influence later childhood outcome, particularly in premature babies (21).

Those measures that go against this framework of care for the newborn and their families should be measured carefully in order to balance the costs for the newborn, and also for the families. Although adherence to contingency plans is critical, some recommendations may be based on fear rather than evidence-based decision-making. It is therefore desirable that contingency plans be periodically modified on the basis of accumulated scientific evidence, and as far as possible, the measures taken during pandemic outbreaks should have as little impact as possible on this family-centered care model while following the guiding principle of cushioning the impact they have on binomial-focused infant-family care.

Importantly, health-workers should rely positively on the contingency plans and help parents to reduce their fear and encourage them to participate in their children's care. Adequate family psychological support and the participation of social workers are essential components of this effort.

Beyond all these threats, in the current coronavirus pandemic outbreak there are opportunities to develop strategies to maintain the excellence of perinatal care. In fact, a stressful environment may have value aspects as it can promote progress and further discussion about care betterment (11).

Telehealth is a growing strategy to reduce the limitations that exist in knowledge and expertise in different areas of the planet, as well as to facilitate telematic contact in situations of restrictions. Telehealth could help in two notable ways by: (a) providing telemedicine-guided assessment to avoid transfers and to take advantage of highly-experienced colleagues, and (b) promoting family involvement with the infant, among relatives that support the labile emotional state of parents, and with social and health workers through a videoconferencing presence at daily rounds. In addition, this tool could be used for remote rounds to enhance family education and neonatal follow-up after discharge (22).

Accepting that every pandemic imposes a learning curve on how to prevent and control the outbreak, we should not overlook the opportunity to resolve epidemiological, organizational, diagnostic, and treatment questions throughout qualitative and quantitative research projects. The follow-up of infected patients and recording of outcomes in databases are essential to improving knowledge and experience for future prevention and treatment strategies (Figure 1).

## Author Contributions

The authors have participated in the conception, writing, and reviewing of the manuscript.

## Conflict of Interest

The authors declare that the research was conducted in the absence of any commercial or financial relationships that could be construed as a potential conflict of interest.
